# Unsupervised Domain Adaptive Corner Detection in Vehicle Plate Images

**DOI:** 10.3390/s22176565

**Published:** 2022-08-31

**Authors:** Kyungkoo Jun

**Affiliations:** Department of Embedded Systems Engineering, Incheon National University, Incheon 22012, Korea; kjun@inu.ac.kr

**Keywords:** license plate recognition, rectification, corner detection, domain adaptation, heatmap

## Abstract

Rectification of vehicle plate images helps to improve the accuracy of license-plate recognition (LPR). It is a perspective-transformation process to project images as if taken from the front geometrically. To obtain the projection matrix, we require the (*x*, *y*) coordinates of four corner positions of plates in images. In this paper, we consider the problem of unsupervised domain adaptation for corner detection in plate images. We trained a model with plate images of one country, the *source domain*, and applied a domain adaptation scheme so that the model is able to work well on the plates of a different country, the *target domain*. For this study, we created a dataset of 22,096 Korea plate images with corner labels, which are source domain, and 6762 Philippines, which are target domain. To address this problem, we propose a heatmap-based corner-detection model, which outperforms existing scalar-regression methods, and an image classifier for mixed image of source and target images for domain adaptation. The proposed approach achieves better accuracy, which is 19.1% improvement if compared with baseline discriminator-based domain adaptation scheme.

## 1. Introduction

**Figure 1:**
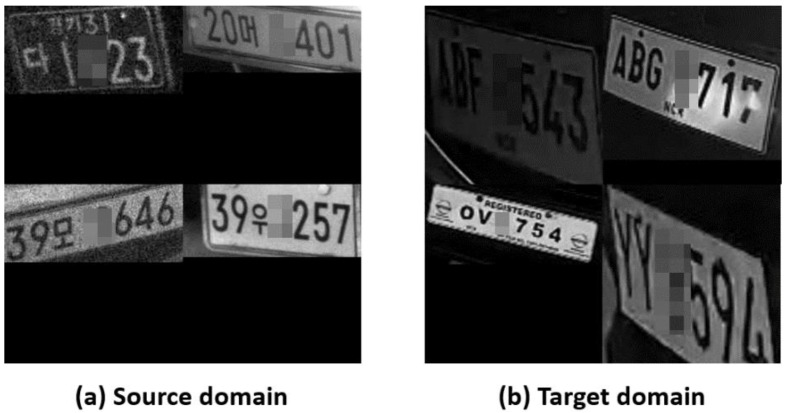
Examples of non-rectified vehicle plates: (**a**) source KR and (**b**) target PH.

License-plate recognition (LPR), in general, consists of two steps [1,2]. In step one, the license plate area is localized, and then in step two, character recognition is performed within the plate area. When the localized plate areas are not properly rectified, as shown in Figure 1, the accuracy of the character recognition degrades. Thus, the warping of plate images as if seen from the straight front would help to improve the performance of LPR. We call this transformation process rectification. Other benefits of the rectification are twofold: (1) it makes the labeling work easier and (2) reduces the dependency on image augmentation during training phase.

The rectification can be easily achieved by perspective transformation [3]. It is to shift one view angle of an object to another view of the same object. This process involves ℋ, a homography 3×3 matrix, as shown in Equation (1):

(1) $$\begin{array}{c} \begin{array}{c} \left[\begin{array}{c} x_{i}^{\prime }\\ y_{i}^{\prime }\\ 1 \end{array}\right]=H\left[\begin{array}{c} x_{i}\\ y_{i}\\ 1 \end{array}\right] \end{array}\\ \begin{array}{c} \left[\begin{array}{c} x_{i}^{\prime }\\ y_{i}^{\prime }\\ 1 \end{array}\right]=\left[\begin{array}{c} h_{11}\\ h_{21}\\ h_{31} \end{array}\begin{array}{c} h_{12}\\ h_{22}\\ h_{23} \end{array}\begin{array}{c} h_{13}\\ h_{23}\\ h_{33} \end{array}\right]\left[\begin{array}{c} x_{i}\\ y_{i}\\ 1 \end{array}\right] \end{array} \end{array}$$

where$\;\left(x_{i},y_{i},1\right)$ and $\left(x_{i}^{'},\;y_{i}^{'},1\right)$ are homogeneous coordinates of a point before and after perspective transformation, respectively. To determine ℋ, we need four pairs of corresponding points of $\left(x_{i},\;y_{i}\right)$ and $\left(x_{i}^{'},\;y_{i}^{'}\right)\;$for i=1…4, which has the following matrix relationship of Equation (2). It represents eight equations to determine the 8 degrees of freedom of ℋ, excluding $h_{33}$, which is fixed to 1 in homogeneous coordinate system. ℋ can then be obtained by applying inverse matrix multiplication to Equation (2).

(2) $$\left[\begin{array}{c} x_{1}^{\prime }\\ y_{1}^{\prime }\\ x_{2}^{\prime }\\ y_{2}^{\prime }\\ x_{3}^{\prime }\\ y_{3}^{\prime }\\ x_{4}^{\prime }\\ y_{4}^{\prime } \end{array}\right]=\left[\begin{array}{cccccccc} x_{1} & y_{1} & 1 & 0 & 0 & 0 & -x_{1}x_{1}^{\prime } & -x_{1}y_{1}^{\prime }\\ 0 & 0 & 0 & x_{1} & y_{1} & 1 & -x_{1}y_{1}^{\prime } & -y_{1}y_{1}^{\prime }\\ x_{2} & y_{2} & 1 & 0 & 0 & 0 & -x_{2}x_{2}^{\prime } & -x_{2}y_{2}^{\prime }\\ 0 & 0 & 0 & x_{2} & y_{2} & 1 & -x_{2}y_{2}^{\prime } & -y_{2}y_{2}^{\prime }\\ x_{3} & y_{3} & 1 & 0 & 0 & 0 & -x_{3}x_{3}^{\prime } & -x_{3}y_{3}^{\prime }\\ 0 & 0 & 0 & x_{3} & y_{3} & 1 & -x_{3}y_{3}^{\prime } & -y_{3}y_{3}^{\prime }\\ x_{4} & y_{4} & 1 & 0 & 0 & 0 & -x_{4}x_{4}^{\prime } & -x_{4}y_{4}^{\prime }\\ 0 & 0 & 0 & x_{4} & y_{4} & 1 & -x_{4}y_{4}^{\prime } & -y_{4}y_{4}^{\prime } \end{array}\right]\left[\begin{array}{c} h_{11}\\ h_{12}\\ h_{13}\\ h_{21}\\ h_{22}\\ h_{23}\\ h_{31}\\ h_{32} \end{array}\right]$$

For example, we are given image A, which contains a non-rectangular shape of a plate P. Let ${\left\{\left(x_{i},y_{i}\right)\right\}}_{i=1}^{4}$ denote the four corner coordinates of P. The corners correspond to left-top, right-top, left-bottom, and right-bottom in order. Then our goal is to generate a new image, B, in which P is transformed into a rectangular-shaped $P^{\prime }$. The perspective transformation enables this process if ℋ is determined. Let ${\left\{\left(x_{i}^{'},y_{i}^{'}\right)\right\}}_{i=1}^{4}$ indicate the four corner positions of $P^{\prime }$ in B. Note that $\left(x_{i}^{'},\;y_{i}^{'}\right)$ is known in advance; they are the predetermined positions where we want to place the transformed plate in B. Therefore, ${\left\{\left(x_{i},y_{i}\right)\right\}}_{i=1}^{4}$ in A is the information we need to find in order to calculate ℋ.

This work proposes a novel deep model that locates four corners ${\left\{\left(x_{i},y_{i}\right)\right\}}_{i=1}^{4}$ from plate images. Inspired by the latest keypoint detection models, our model is designed to output a set of four heatmaps. The heatmaps correspond to each one of four corners. They embed 2D Gaussian blobs, which represent the probabilities of corner locations. The requirements for the rectification are for real time and have resource efficiency. Considering the entire LPR process, less emphasis is put on the rectification compared with the localization and the recognition. It means that the rectification component should use small and shallow models that consume less computing time and resources.

In this work, another challenge is that we do not assume that training and test data are collected from an identical distribution. The training data are Korean plates, and the test data are Philippines plates. Such a distribution discrepancy has been observed to cause a performance drop in various works. The Korean and Philippines plates are different from each other in regard to shapes, sizes, and characters. Hence, a corner-detection model trained with the Korean plates would not work well with the Philippines. Thus, it is desirable to develop methods that can adapt corner-detection models to unseen domains, which are visually different from the training data. We address this cross-domain corner-detection problem by developing algorithms to transfer knowledge from a labeled source domain of Korean plate images to an unlabeled Philippines.

Domain adaptation techniques [4] aim at reducing the domain gap between the source and target domain. In particular, adversarial discriminative domain adaptation methods [5] have been proposed to improve performance by discouraging discrimination capability between source and target domain data. Our work proposes a different type of data discrimination. We trained the discriminator to tell how source and target domain images are cut and pasted in a new image. This task is more challenging than binary domain discrimination. The discriminator trained with this scheme becomes harder to be deceived; as a result, the feature extractor is guided to capture more domain-invariant plate features. 

The contribution of this work can be summarized as follows.

1. We proposed a corner-detection model which outputs a likelihood heatmap. It outperforms existing models based on scalar regression. In addition, we created a new dataset consisting of 22,096 KR plate images and 6762 PH plate images.
2. We presented a novel domain adaptation based on an adversarial discriminative method. It is peculiar in the sense that the discriminator is required to distinguish how Korean plates and target Philippines plates are mixed.
3. We conducted experiments on various corner-detection tasks and validated that our method can bring performance gains by 19.1% if compared with baseline discriminator-based domain adaptation method.

This paper is organized as follows: Section 2 surveys existing works related with our work in regard to subjects such as rectification and domain adaptation, Section 3 presents our proposed model and domain adaptation scheme, Section 4 shows evaluation results, and Section 5 concludes the paper.

## 2. Related Work

**Corner Detection:** Finding the four corner positions of vehicle plates for the rectification purpose can be considered as a keypoint detection problem. CNN-based methods [6,7] use features or latent vectors extracted through a sequence of convolution layers to regress corner coordinates. Instead of regressing corners, the methods [8,9] tried to directly generate rectified images. However, these methods are impractical considering the real-time constraint of LPR tasks and degradation of output quality; the image generation models are, in general, computation intensive, and the output images often contain blurred parts. 

Recently, Vision Transformer (ViT) [10] is proposed, which depends on a representative transformer model for natural language processing. ViT converts the input image into a sequence of image patches and extracts context information through a self-attention structure with multi-heads. ViT was used to unwarp document images with geometric distortion [11]. The self-attention mechanism, a core element of ViT, is limitedly used only for low-resolution image input due to computational complexity. Moreover, there have been questions about whether it will be effective for tasks that require high input resolution, such as detection or segmentation. For the detection task, a model has been proposed that uses ViT as a backbone [12] with limited success.

**Keypoint Detection**: 2D keypoint detection is actively studied for its wide applicability in computer vision tasks. Most of the works are based on heatmap-output networks. A multi-resolution framework was proposed that generates heatmaps representing per-pixel likelihood for keypoints [13]. Hourglass networks for heatmaps of human body keypoints were developed for pose estimation [14]. The heatmaps from deconvolutional-layer-added ResNet [15] was utilized for human pose determination.

**Domain Adaptation and Keypoint Detection**: When domain shift occurs, most deep models experience performance deterioration because of unseen data. One of active works to deal with such degradation is domain adaptation. It has been actively studied in computer vision. Earlier works have mostly focused on image classification. Recent works have widened its application areas, aiming to improve the domain adaptability of deep neural networks, including References [16,17,18,19,20]. We focus on the keypoint detection task, which has been studied lately compared to other areas.

Some previous works have investigated domain adaptation in keypoint detection. Most works are related with 3D keypoints detection. A weakly supervised method using depth images was proposed in Reference [21], domain adaptation with a 3D geometric constraint-aware loss was studied in Reference [22], and prediction regularization for unlabeled target domain in 3D keypoints detection has been enforced by view-consistency during domain adaptation [23]. Our problem setup is different from those works in that we considered 2D image keypoint detection and the domain adaptation.

## 3. Corner-Detection Model and Domain Adaptation

**Figure 2:**
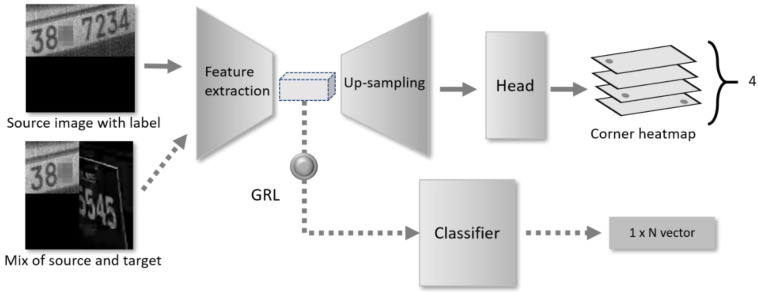
The architecture of the proposed corner-detection model using adversarial mixed image discriminator.

An overview of our network is shown in Figure 2. It consists of two main parts; the upper part of Figure 2 is the corner-detection components, and the lower part is a classifier, which plays a role as the domain adaptation component in our scheme. The classifier distinguishes input image as one of N label types by giving out one-hot vector of length N. The details about the classifier and its output vector are discussed later.

**Corner-Detection Model**: The corner-detection model extracts a set of likelihood heatmaps about the corner positions from input images. The feature extractor is the backbone of ResNet18 pretrained on ImageNet, followed by the up-sampling component using deconvolution layers. The head predicts four corner locations, namely $p_{1},\;p_{2},\;p_{3},\;\mathrm{and}\;p_{4}$ for top-left, top-right, bottom-left and bottom-right, respectively, using heatmap $f\left(p_{k}\right)\in R^{H^{\prime }\times W^{\prime }}$ for corner point$\;p_{k}$. The heatmaps contain 2D Gaussian blobs centered at the predicted corner locations. The corner position $\left(x_{k},y_{k}\right)$ is determined by the following:

(3) $$J\left(f_{\;}\left(p_{k}\right)\right)=arg\;\underset{x,y}{\max}f{\left(p_{k}\right)}_{x,y}{{}_{\;}}_{\;}$$

**Figure 3:**
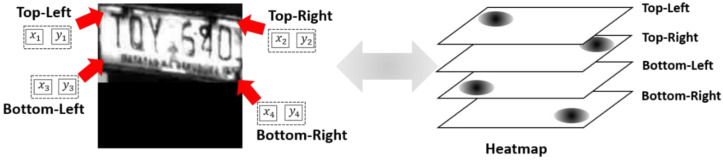
Corner positions and corresponding heatmaps. Each heatmap is responsible for only one corner.

Figure 3 shows the corner locations, $p_{k}$, of images and corresponding heatmaps. The four heatmaps correspond to each of the corner positions.

Let us denote $H_{i}\left(p_{k}\right)\in R^{H^{\prime }\times W^{\prime }}$ as the heatmap label of the corner point, $p_{k}$, for the *i*-th training image, with 2D Gaussian blob centered on the ground truth coordinate of $p_{k}$. Then we can use the cross-entropy loss, and the heatmap loss can be written as follows:

(4) $${ℒ}_{H}=-\underset{i,k}{\sum }\left[H_{i}\left(p_{k}\right)\log f_{i}(p_{k})+\left(1-H_{i}\left(p_{k}\right)\right)\log\left(1-f_{i}\left(p_{k}\right)\right)\right]$$

**Domain Classifier**: The domain classifier in our network is the domain adaptation component to align the feature representation distributions on image level. The image-level representation refers to the feature map from the feature extractor. To reduce the difference between the domain distributions on the image level, the classifier consists of repetitions of a sequence of a convolutional layer, batch normalization, and ReLU activation, followed by an adaptive average pooling and a fully connected layer.

For the classifier training, images with Korean and Philippines plates cut in half and put together side-by-side are used. The classifier predicts the cut-and-paste label for the images. The output dimension of the classifier, 1×N, in Figure 2 indicates the number of classification classes. Let us denote $D_{i}$ as the label of the *i*-th training image, $D_{i}=0$ for Korean on the left half and Philippines right half, and $D_{i}=1$ for the opposite. Therefore, in the current settings, we set N=2. When devising new mix style for classification in the future, we can add new label types by increasing N, which generally helps to improve the adaptation capability of the proposed model.

The benefits of this image mix-up are twofold: (1) the mix-up is generally harder dataset. Thus, the domain adaptation phase leads to more robust results. The performance comparison results when using source and target images separately proves this claim in the following section, and (2) the feature extractor is guided to eliminate the character-specific features in the feature maps, which helps to maximize the classification loss in the adversarial training phase. As a result, the feature maps are more likely to contain more plate-shape-related information.

By denoting the output of the classifier as $c_{i}$ and using the cross-entropy loss, the domain adaptation loss can be written as follows:

(5) $${ℒ}_{DA}=-\underset{i}{\sum }\left[D_{i}\log c_{i}+\left(1-D_{i}\right)\log\left(1-c_{i}\right)\right]$$

To align the domain distributions, we should simultaneously optimize the parameters of the classifier to minimize the ${ℒ}_{DA}$ and also optimize the parameters of the feature extractor to maximize ${ℒ}_{H}{}_{\;}$. For the implementation, we use the gradient reverse layer (GRL) [15], whereas the ordinary gradient descent is applied for training the classifier. The sign of the gradient is negated when passing through the GRL layer to optimize the feature extractor.

The overall network of Figure 2 is involved only in the training phase. During inference, one can exclude the domain adaptation components, such as the classifier and the GRL, and simply use the corner-detection model with adapted parameters.

## 4. Experiments

**Experiment Setup**: The dataset consists of two domains: Korean plate images with a total of 22,096 and Philippines with a total of 6762 images. Following the common terminology in domain adaptation, we refer to the Korean images as source domain, denoted by KR, and to the Philippines images as target domain, PH. All of the images have the same dimension of 416×416 and are gray with one channel. For each domain, we split the images into 8:2 ratio for training and test purpose. The corner prediction model outputs are the heatmaps of the dimension of 104×104.

We construct the ground truth heatmaps from the labels, which have the normalized coordinates of the four corner positions. The heatmaps have 2D Gaussian blobs centered at the corner locations. We determine the blob size by standard deviation, σ.

We finetune ResNet18 of the feature extractor pretrained on ImageNet. The up-sampler and the heatmap head are trained from scratch, with an initial learning rate of 1 × 10^−3^. We adopt mini-batch SGD with a momentum of 0.9 and a batch size of 64. The learning rate is adjusted by $\eta_{p}=\eta_{0}\;\alpha^{p}$, where p is the training steps, $\eta_{0}=1^{-3}$, and α=0.95. Our model is trained for 500 iterations. We chose the optimal model state guided by the highest performance on the validation sets of KR and PH.

Four models are involved in the experiments, as listed in Table 1. The sizes and the parameter numbers of the models are shown. Conv. and MNet. are from the existing works [6,7]. Those models work in a regression way, predicting normalized coordinates of the corners. We do not apply any domain adaptation scheme to those models. We train them by using only the source-domain data. Classic-DA is the corner-detection model with well-known domain adversarial discriminator [24]. The architecture of this model is similar to ours except that the discriminator is used instead of the classifier. Proposed-DA is our model. It reduces the number of parameters slightly compared with Classic-DA because the classifier size is smaller than the discriminator of the Classic-DA.

Regarding training data, Conv. and MNet. use only the source images and corresponding labels because the target images have no label information that is required for supervised training. For Classic-DA and Proposed-DA training, both the source and target images are used. Moreover, we train Proposed-DA with only source images and only target images, denoted by Source-Only, and Target-Only, respectively. These are used as baselines to assess the effectiveness of the proposed method.

As evaluation metric, we measure error distances between predicted corner positions and the ground truth are measured in pixel units. The errors for the four corner positions, Left-Top, Right-Top, Left-Bottom, and Right-Bottom, respectively, are measured separately. We run two experiments, one using source KR as the test dataset, and the other with target PH as the test dataset. 

**Experimental Results**: Table 2 summarizes the evaluation results from testing with source KR dataset. Since the test dataset includes no target images, it is not related to domain adaption. Its purpose is to obtain the baseline performance for comparison and evaluate the heatmap regression of the proposed model. The downward arrows in the table mean that the lower the values are, the more accurate the prediction is. We observe that all the heatmap-based methods of Source-Only, Classic-DA, and Proposed-DA show less error than non-heatmap based methods of Conv. and MNet. Thus, note that the heatmap scheme is more effective for regressing the corner locations than scalar value regression.

Observing the results of the heatmap based Source-Only, Classic-DA, and Proposed-DA, we find that they show similar errors. Recall that the training of Source-Only is different from Classic-DA and Proposed-DA; its training uses only the source KR dataset, while two models use both KR and PH dataset. This finding verifies that the target images included in training have little influence on the proposed domain adaptive model. The model is able to maintain accuracy about the source domain, even though it is trained to prepare for domain adaptation. Interestingly, Source-Only slightly outperforms the other two methods. It is obvious because the models trained and tested with same domain dataset perform best.

The test results for the target PH dataset are summarized in Table 3. In this experiment, we use the model of Target-Only, which is trained and tested only with the target dataset. It is no surprise that Target-Only shows the best results. It is because the model is trained and tested in the same domain. We present its results here just for providing an example of the best accuracy that the domain adaptive schemes should achieve.

These experiment results for the source and target dataset highlight that the domain adaptation methods generalize better than the Source-Only model. The proposed method of Proposed-DA is in general more robust to domain shift than the Classic-DA. This suggests that the classifier for mixed images was more effective for decreasing the domain gap between the two distributions than the image discriminator. It is worth noting that the proposed method exhibits a drastic performance improvement of 19.1% on average if compared with the results of the Class-DA.

**Figure 4:**
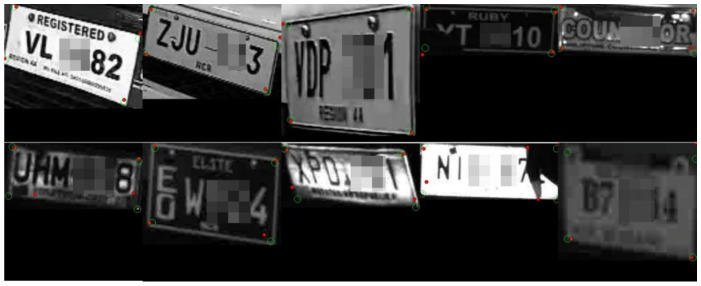
Qualitative evaluation results of the proposed domain adaptation scheme.

Figure 4 shows the qualitative prediction results of the proposed domain adaptive corner detection. We show the ground-truth corners as green circles and the predicted corner locations as the red dots. Some of the characters on the plates are intentionally blurred for privacy protection. In general, the proposed method is able to locate most corners of unseen PH plates. As can be noted, the plate images with clear and distinctive edges show the successful prediction results. Regarding the incorrect results, possible reasons are as follows: (1) plates are partly broken (second row, fourth from left), (2) the boundary lines are blurred with background (second row, first from right), and (3) defects on the plates are present (second row, first from left).

**Experiments with deeper backbone networks**: We also evaluated with deeper backbones such as ResNet34, ResNet50, etc., to assess its effect on the performance. In the current architecture, ResNet18 is adopted as the backbone. As mentioned in Section 1, the rectification component has a limitation in regard to adopting heavier models due to real-time and resource constraint. Since ResNet18 is smaller and faster than other models, it helps LPR to meet real-time requirements. In this experiment, we evaluated the tradeoff between speed and accuracy by employing deeper networks.

For comparison among deeper backbones, we tested with ResNet34, ResNet50, ResNet101, and ResNet152, along with the original backbone of ResNet18. We prepared a set of Proposed-DA models, each with those different backbone networks. The models were trained with the same dataset and parameters until the loss decrease stabilizes. The evaluation used the same test datasets: source KR test dataset and target PH dataset. We used two comparison metrics: the corner errors and the inference time.

As shown in Table 4 for the source KR and Table 5 for the target PH, concerning the prediction errors, the deeper backbones such as ResNet50 and higher models outperform shallow backbones such ResNet18 in both test datasets. However, the error improvements are not substantial, indicating that ResNet18 is sufficiently accurate to be used in real-time environment. Interestingly, the deepest models of ResNet101 and ResNet152 show larger errors than the shallower ResNet50. The training difficulties often encountered with deep and huge models are presumed to cause such lower accuracy.

Table 6 shows average elapsed time (ms) required to predict four corners when given a single image to the Proposed-DA. The images have the dimension of 416 × 416. We measure the times in two separate environments: CPU and GPU. The reason to consider CPU for comparison is that LPR in real-world applications often runs on platforms without GPU. The times are averaged over source KR and target PH test dataset images. As expected, the ResNet18-based model runs fastest, and the deeper models are almost 10 times slower. The results imply that the deeper networks are not suitable for real-time LPR applications, which should finish within, at most, 500 ms, including localization and recognition. In this sense, ResNet18 is a proper selection for the backbone of our proposed model.

## 5. Conclusions

We considered the problem of unsupervised domain adaptation for plate corner detection. To conduct our study, we prepared a new dataset consisting of 22,096 KR plate images and 6762 PH plate images, which we can provide under certain agreement. Our main contributions are twofold; we proposed a heatmap-based corner-detection model, which is better than the existing scalar-regression-based methods, and the domain adaptation using mixed image classifier, an effective approach for cross-domain object detection. Our approach is validated on experiments, and our method outperforms the baseline discriminator-based domain adaptation scheme with 19.1% improved accuracy, as well as existing corner-detection methods, thus demonstrating its effective for corner detection in a domain-shift environment.

Although our method can achieve compelling results in the target PH dataset, the results are far from generalization. It may fail when testing with other license plates of different countries. Future work may extend the proposed domain adaptation scheme to become more general, and to other application scenarios beyond license plates and will explore the possibility of combining rotation and flip techniques in adaptation steps to improve performance on the current test dataset. We hope that the proposed dataset will motivate research on this topic and our domain adaptation scheme will serve as a strong baseline for future works.

## Figures and Tables

**Table 1:** The sizes and number of parameters of the modes used in the experiments.

Model	Model Size (MB)	No. of Parameters (K)
Conv.	352	92,203
MNet.	17	4150
Classic-DA	64	16,563
Proposed-DA	64	15,963

**Table 2:** Error distances in pixel for KR source domain images. The least errors are in bold.

Method	Left-Top	Right-Top	Left-Bottom	Right-Bottom	Avg.
Conv.	9.89	10.50	11.65	8.15	10.9
MNet.	6.78	7.19	8.07	5.30	6.8
Source-Only	**5.2**	**5.8**	6.1	**4.4**	**5.4**
Classic-DA	5.74	6.13	**6.08**	4.5	5.6
Proposed-DA	5.69	5.90	6.27	4.66	5.6

**Table 3:** Error distances in pixel of the models for PH target domain images.

Method	Left-Top	Right-Top	Left-Bottom	Right-Bottom	Avg.
Conv.	25.84	31.69	37.23	32.43	31.8
MNet.	18.57	21.12	19.05	25.34	21.0
Target-Only	6.51	8.09	9.00	8.53	8.03
Source-Only	18.0	20.2	24.1	24.0	21.6
Classic-DA	17.08	20.4	21.2	**20.8**	19.9
Proposed-DA	**10.34**	**13.36**	**15.39**	20.96	**15.01**

**Table 4:** Errors for source KR dataset of Proposed-DA with different backbone networks.

Backbone	Left-Top	Right-Top	Left-Bottom	Right-Bottom	Avg.
ResNet18	5.69	5.90	6.27	4.66	5.6
ResNet34	5.50	5.80	5.97	4.57	5.46
ResNet50	**5.30**	**5.50**	**5.80**	**4.42**	**5.25**
ResNet101	5.53	5.84	6.13	4.70	5.55
ResNet152	5.52	5.90	6.18	4.69	5.57

**Table 5:** Errors for target PH dataset of Proposed-DA with different backbone networks.

Backbone	Left-Top	Right-Top	Left-Bottom	Right-Bottom	Avg.
ResNet18	10.34	13.36	15.39	20.96	15.01
ResNet34	10.15	12.97	15.22	18.45	14.19
ResNet50	**10.01**	**12.80**	**14.80**	**17.90**	**13.87**
ResNet101	10.04	13.01	15.10	19.03	14.29
ResNet152	10.03	13.04	15.05	19.22	14.33

**Table 6:** Average elapsed time (ms) for a single image corner prediction of Proposed-DA.

Backbone	Core-i7 CPU	Nvidia GTX 1660 Super (6G)
ResNet18	64.5 ms	8.8 ms
ResNet34	109.3 ms	17.4 ms
ResNet50	540.8 ms	52.1 ms
ResNet101	1100.9 ms	98.3 ms
ResNet152	1645.1 ms	120.7 ms
